# Immune infiltration and immunophenotyping in atrial fibrillation

**DOI:** 10.18632/aging.204470

**Published:** 2023-01-04

**Authors:** Yuqing Tian, Shiying Liu, Yanan Zhang, Jiefu Yang, Peiyao Guo, Hongchao Zhang, Xue Yu, Tong Zou

**Affiliations:** 1 Department of Cardiology, Affiliated Hospital of Panzhihua University, Panzhihua 617000, Sichuan, P.R. China; 2 Beijing Hospital, National Center of Gerontology, Institute of Geriatric Medicine, Chinese Academy of Medical Sciences and Peking Union Medical College, Beijing 100730, P.R. China; 3 Department of Plastic Surgery, Affiliated Hospital of Panzhihua University, Panzhihua 617000, Sichuan, P.R. China; 4 Department of Critical Care Medicine, The Affiliated Hospital of Qingdao University, Qingdao 266000, Shandong, P.R. China; 5 Department of Cardiology, Beijing Hospital, National Center of Gerontology, Institute of Geriatric Medicine, Chinese Academy of Medical Science, Beijing 100730, P.R. China; 6 Peking University Fifth School of Clinical Medicine, Beijing Hospital, Beijing 100730, P.R. China

**Keywords:** atrial fibrillation, immune infiltration, Gene Expression Omnibus, signature gene, inflammatory response

## Abstract

Atrial fibrillation (AF) is a relatively common arrhythmia in clinical practice. Although significant progress has been achieved in the treatment of AF and its associated complications, research on AF prevention lags behind, mainly due to the lack of a deep understanding of AF pathogenesis. In recent years, as our knowledge has grown, the role of the inflammatory/immune response in the occurrence and progression of AF has gradually gained attention. In this paper, based on existing gene expression data in the Gene Expression Omnibus database, a detailed description of immune infiltration status in AF is presented using a series of analytical methods, including differential analysis, Gene Ontology categorization, Kyoto Encyclopedia of Genes and Genomes enrichment analysis, and weighted gene coexpression network analysis, and analysis tools such as CIBERSORTx and Cytoscape. Several new AF/immune infiltrations–related signature genes were identified, and the AF/immune infiltration pathology was classified based on these immune signature genes, thus providing novel insights into the pathogenesis of AF based on the inflammatory response.

## INTRODUCTION

Atrial fibrillation (AF) is a common arrhythmia during clinical diagnosis and treatment in cardiology departments. Due to the rapid advancement of surgical instruments and cardiac drugs, significant progress has been achieved in the treatment of AF and its complications (tachyarrhythmia, thrombosis, heart failure, etc.) [1]. According to a systematic review including population studies worldwide, the number of AF patients globally was estimated to be 33.5 million in 2010 [2–4]. However, our understanding of the mechanism of AF occurrence and persistence is still inadequate.

Many case–control studies have demonstrated that the levels of inflammatory markers such as C-reactive protein (CRP), interleukin-6 (IL-6), IL-8, and tumor necrosis factor-α (TNF-α) in the AF patient population are significantly higher than those in the sinus rhythm population [5–10]. Additionally, the CRP level can be used to predict new-onset AF [11–13]. These results indicate a certain correlation between the inflammatory response and the occurrence of AF. Notably, in a canine sterile pericarditis model, anti-inflammatory treatment significantly reduced the incidence of aseptic inflammation-induced AF [14], which may suggest that the inflammatory response is the initiating factor in AF.

AF induced by inflammation can stimulate the body to generate new inflammatory responses and initiate atrial remodeling, resulting in AF maintenance [1]. AF patients have higher levels of inflammatory markers than previous AF patients who have recovered sinus rhythm [15, 16]. After continuous fast atrial pacing in dogs (pacing time >1 week), peripheral blood CRP levels were significantly increased, the atrial effective refractory period was shortened, and AF susceptibility was increased [17], indicating that AF may trigger new inflammatory responses, which likely result in AF persistence [17].

In summary, although the inflammatory response and AF are strongly correlated, the underlying mechanisms remain poorly understood. With the widespread application of second-generation sequencing technology and immune infiltration analysis technology, more powerful research tools are available to solve this problem. Therefore, this study (Supplementary Figure 2) was conducted to screen AF/immune infiltration–related differentially expressed genes (DEGs) based on existing gene expression data from the Gene Expression Omnibus (GEO) database. On the basis of these DEGs, the weighted gene co-expression network analysis (WGCNA) algorithm was used to identify key modules. Finally, hub genes associated with the AF/inflammatory response were further screened, and their possible role in the development of AF is discussed.

## MATERIALS AND METHODS

### Data download

GEOquery [18] was used to download four sets of data: GSE115574, GSE41177, GSE79768, and GSE2240. GSE2240 was used as the verification dataset. Probes corresponded to genes, and the median value of the probes for the same gene was used for analysis. The GSE115574, GSE41177, and GSE79768 datasets were pooled. Batch differences (Supplementary Figure 1) were eliminated using the ComBat function of the sva package [19], and then the gene expression levels were homogenized using the limma package [20].

### Immune cell infiltration

The gene expression data of the samples were uploaded to CIBERSORTx (https://cibersortx.stanford.edu/)). Bulk-mode batch correction and absolute mode were selected to analyze the level of immune infiltration, and the absolute score indicating the level of infiltration was obtained for each sample. The median absolute score of all samples was calculated. An absolute score greater than or equal to the median value indicated high infiltration, and an absolute score less than the median indicated low infiltration.

### Analysis of DEGs

The limma [20] package was used to analyze DEGs in a high-infiltration group, a low-infiltration group, an AF group, and a sinus rhythm (SR) group, and the intersections of the DEGs of pairs of groups were obtained. *P*<0.01 was considered indicative of a significant difference.

### Enrichment analysis

The clusterProfiler [21] package of R was used for Gene Ontology (GO) and Kyoto Encyclopedia of Genes and Genomes (KEGG) pathway enrichment analysis of DEGs.

### WGCNA

We used WGCNA to construct a weighted co-expression network of DEGs. Based on traits of interest, the module with the smallest *P* value and the highest correlation was selected as the key module.

### Screening of hub genes

Genes that were most relevant to a trait (gene significance (GS) ≥0.3) and a module (module membership (MM) ≥0.7) among the key WGCNA modules were selected as the hub genes in the modules. All the genes in the key modules were input into the STRING website (https://string-db.org/). The protein–protein interaction (PPI) network was obtained and then plotted using Cytoscape software. Genes with a high degree of gene connectivity (≥30) were selected as key genes in the PPI network. The final set of hub genes was obtained by taking the intersection of the WGCNA key module genes and PPI network key genes.

### Analysis of clinical characteristics of hub genes

The age and sex information of each sample was used for grouping. The expression levels of the hub genes in different groups were analyzed. The median age was set as the dichotomizing line.

### Hub gene validation

The expression levels of the hub genes were verified in GSE165838.

### Unsupervised clustering analysis

Based on the expression levels of the hub genes, the best number of classifications was determined by determining the best sum of squared error (SSE) inflection point, and the samples were classified using k-means clustering combined with t-distributed stochastic neighbor embedding dimensionality reduction. Differences in immune infiltration and the expression levels of hub genes between different types were investigated.

### Data availability statement

The original contributions presented in the study are included in the article/supplementary material, and further inquiries can be directed to the corresponding author/s.

## RESULTS

### Data set selection and content introduction

The AF and SR datasets of GSE115574, GSE41177, GSE79768, and GSE2240 were downloaded from the GEO database (Table 1). GSE2240 was used as the verification dataset. The other three datasets were combined to obtain the expression data of 21,502 genes in 123 samples, including 74 AF and 49 SR samples.

**Table 1:** AF and SR datasets from the GEO database.

**Dataset**	**Platform**	**Atrial fibrillation**	**Sinus rhythm**
GSE115574	GPL570	28	31
GSE41177	GPL570	32	6
GSE79768	GPL570	14	12
GSE2240	GPL96	10	20

### The difference of immune cell infiltration is shown

**Figure 1:**
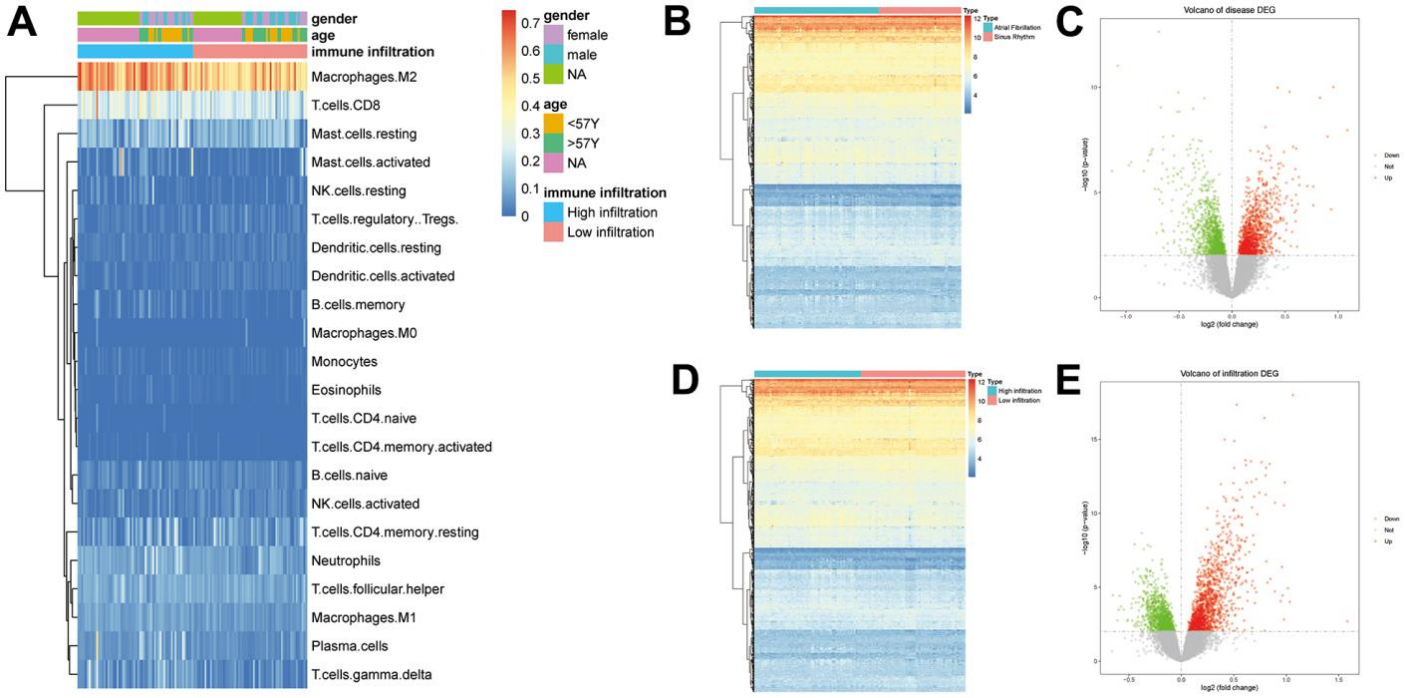
(**A**) Results of the immune infiltration analysis (p>0.05, *: p<0.05, **: p<0.01, ***: p<0.001, ****: p<0.0001). (**B**) Volcano plot of DEGs in AF and SR. (**C**) Heat map for the expression of the 2379 DEGs in AF. (**D**) Volcano plot of DEGs in immune infiltration. (**E**) Heat map for the expression of the 2607 DEGs in immune infiltration.

According to the gene expression data, the immune cell infiltration level was analyzed using CIBERSORTx. We marked the differences of immune cells between the two groups, and the results are shown in the Figure 1A. Each square represents the infiltration level of immune cells in the sample.

### Differential analysis strategy and differential analysis results

*P*<0.01 was used as the threshold to define a DEG. A total of 2379 DEGs were identified in the 74 AF samples and the 49 SR samples, 1213 of which were upregulated, while 1167 were downregulated (Figure 1B, 1C). A total of 2607 DEGs were related to immune infiltration, 1432 of which were upregulated, while 1184 were downregulated (Figure 1D, 1E). The intersection of these two groups included 586 DEGs.

### GO and KEGG enrichment analysis

**Figure 2:**
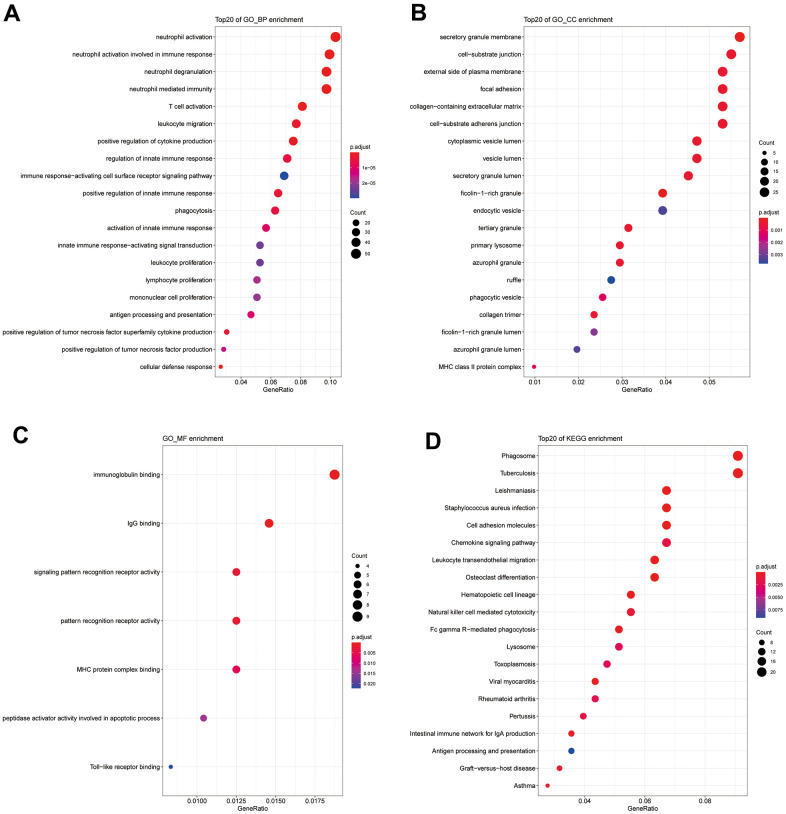
(**A**) GO_BP enrichment results for DEGs. (**B**) GO_CC enrichment results for DEGs. (**C**) GO_MF enrichment results for DEGs. (**D**) KEGG enrichment results for DEGs.

Pathway enrichment analysis of the 586 shared DEGs was performed (Figure 2, P_FDR<0.05). As shown in Figure 2, the GO categories of biological process (BP) and cell component (CC) and the KEGG pathways are shown only with the 20 most significant results. Most were related to immune responses (e.g., the regulation of the innate immune response), immune cells (e.g., T cell activation, leukocyte migration), and immune activities (e.g., chemokine signaling, immunoglobulin binding).

### Construction of WGCNA co-expression network

**Figure 3:**
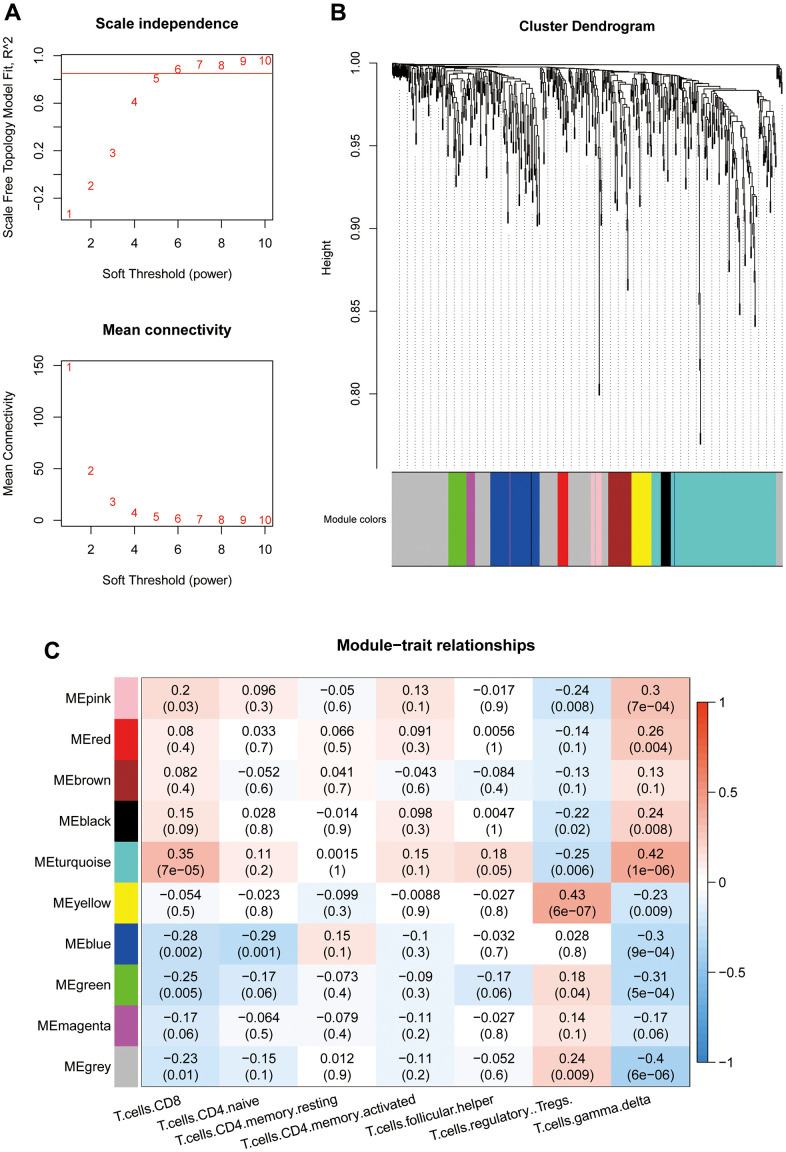
(**A**) Threshold selection for WGCNA network construction (β=6). (**B**) WGCNA network module classification (mergeCutHeight=0.25. The minimum module size is 10.). (**C**) Association between module eigenvectors and T cells. The first rows in each block are the correlation coefficients. Red indicates a positive correlation, and blue indicates a negative correlation. P values are provided in parentheses in the second row.

WGCNA was performed on the 586 shared DEGs (The soft-threshold β=6. The minimum module size is 10. We merged similar modules, mergeCutHeight=0.25. We use Person correlation when we associate features with modules.). A scale-free network was constructed (R^2^=0.8850) (Figure 3A). Hierarchical clustering was performed to divide the network into modules. A total of 10 modules were obtained. In the figure, gray indicates no modules included (Figure 3B). The numbers of genes in each module are shown in Table 2. For several key T cell-related immune modules (*P*<0.05 with a correlation) (Figure 3C), gamma delta cells had a strong correlation with each module. The turquoise module, representing the smallest *P* value and the highest correlation, was selected as the key module and contained a total of 172 genes.

**Table 2:** WGCNA module statistics.

**Module**	**Number of genes**
Pink	15
Red	16
Brown	35
Black	16
Turquoise	172
Yellow	30
Blue	73
Green	27
Magenta	14
Gray	188

### Screening methods and results display of hub genes

**Figure 4:**
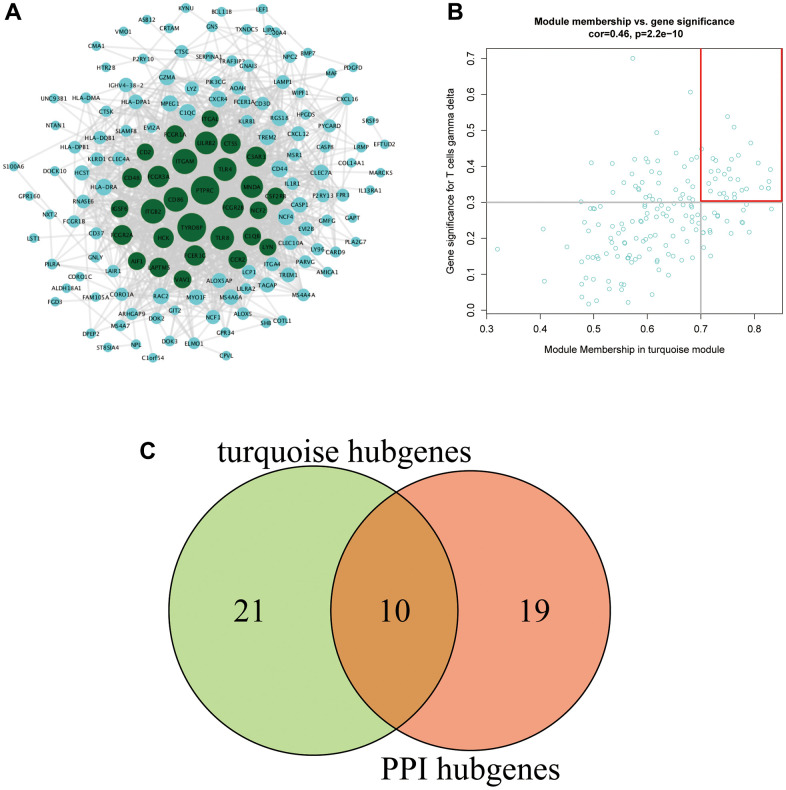
(**A**) PPI network of the turquoise module. The gene node size in the network reflects the degree of nodes, with a higher degree of gene connectivity corresponding to a larger font with which the gene is written. Green represents genes with a connectivity degree ≥30 (a total of 29 genes). (**B**) The key genes in the turquoise module. (**C**) Venn diagram of the key genes.

The 172 genes in the turquoise module were input into the STRING website to construct a PPI network. A total of 29 genes with a connectivity of ≥30 were selected as hub genes (Figure 4A). A total of 31 genes with MM ≥0.7 and GS ≥0.3 were selected as hub genes in the turquoise module (Figure 4B). Ten key genes were obtained after the intersection of key genes of the PPI and key genes of the turquoise module: *CTSS*, *NCF2*, *MNDA*, *CCR2*, *TYROBP*, *LAPTM5*, *IGSF6*, *PTPRC*, *AIF1*, and *ITGAL* (Figure 4C).

### Correlation analysis between hub genes and clinical features

**Figure 5:**
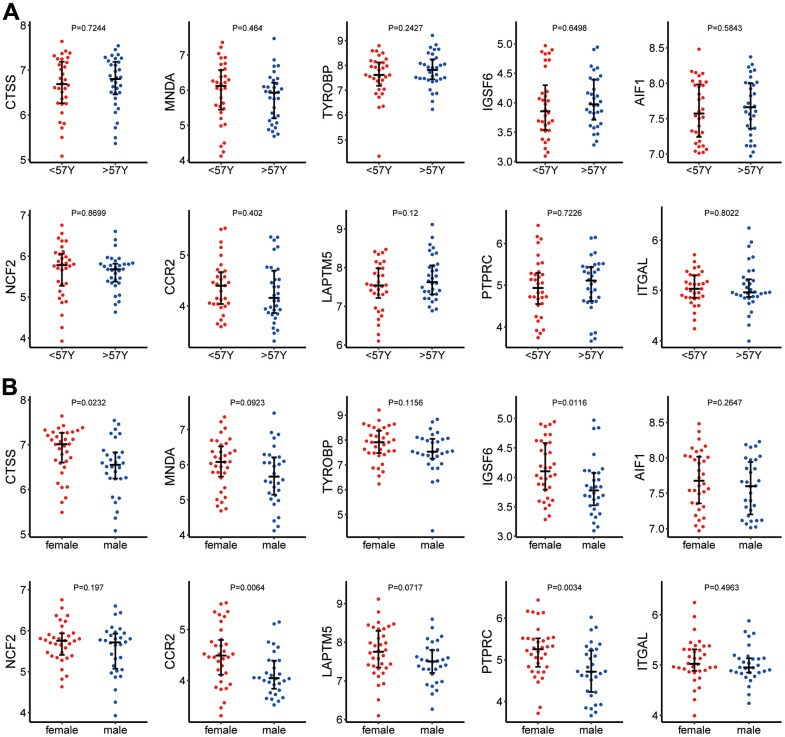
(**A**) Hub gene expression in different age groups. (**B**) Hub gene expression in different sexes.

Among the 123 samples, 64 samples from GSE41177 and GSE79768 contained sex and age information. The median age (57 years) was used as the dichotomizing line for age grouping. Figure 5A shows that hub genes had no significant differences in expression between age groups. Figure 5B shows the expression of hub genes in different sexes. The expression levels of *CTSS*, *IGSF6*, *CCR2*, and *PPTRC* were significantly higher in females than in males.

### Hub genes validation

**Figure 6:**
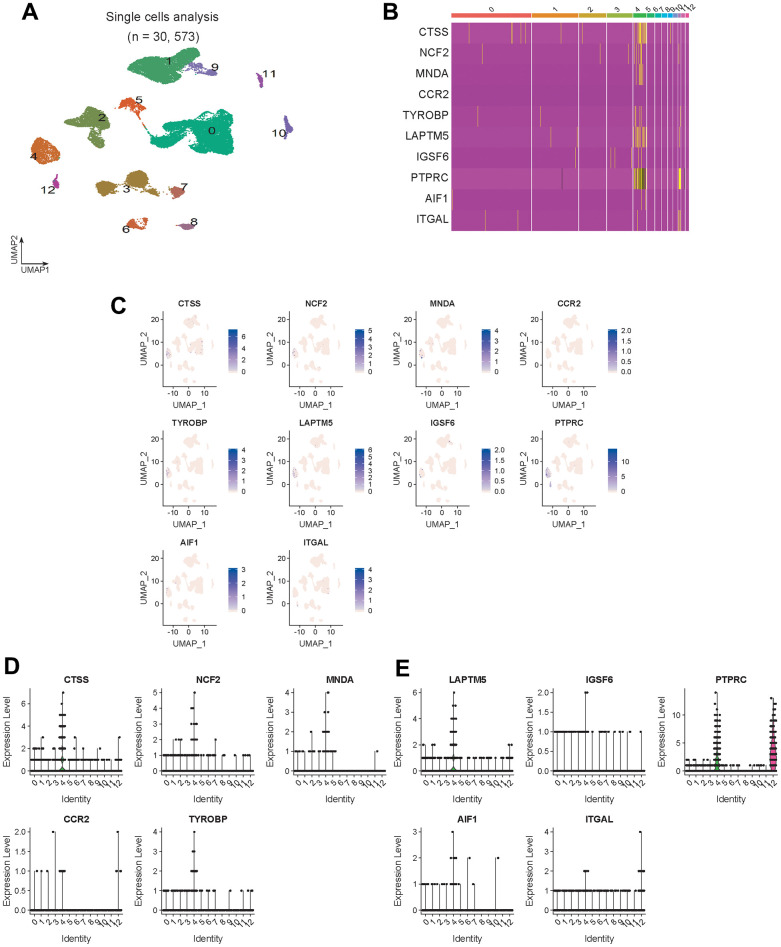
**Hub gene expression validation.** (**A**) Visualized cell cluster map of 30,573 single cells. (**B**) Heat map of 10 hub genes. (**C**) Expression distribution of 10 hub genes in the visual cell cluster. (**D**, **E**) Violin map of 10 hub genes showing the expression distribution of genes in different cell types.

The Seurat function was used to analyze GSE165838, and the expression distribution of 10 hub genes in the scRNA dataset was visualized (Figure 6).

### Unsupervised clustering of hub genes

**Figure 7:**
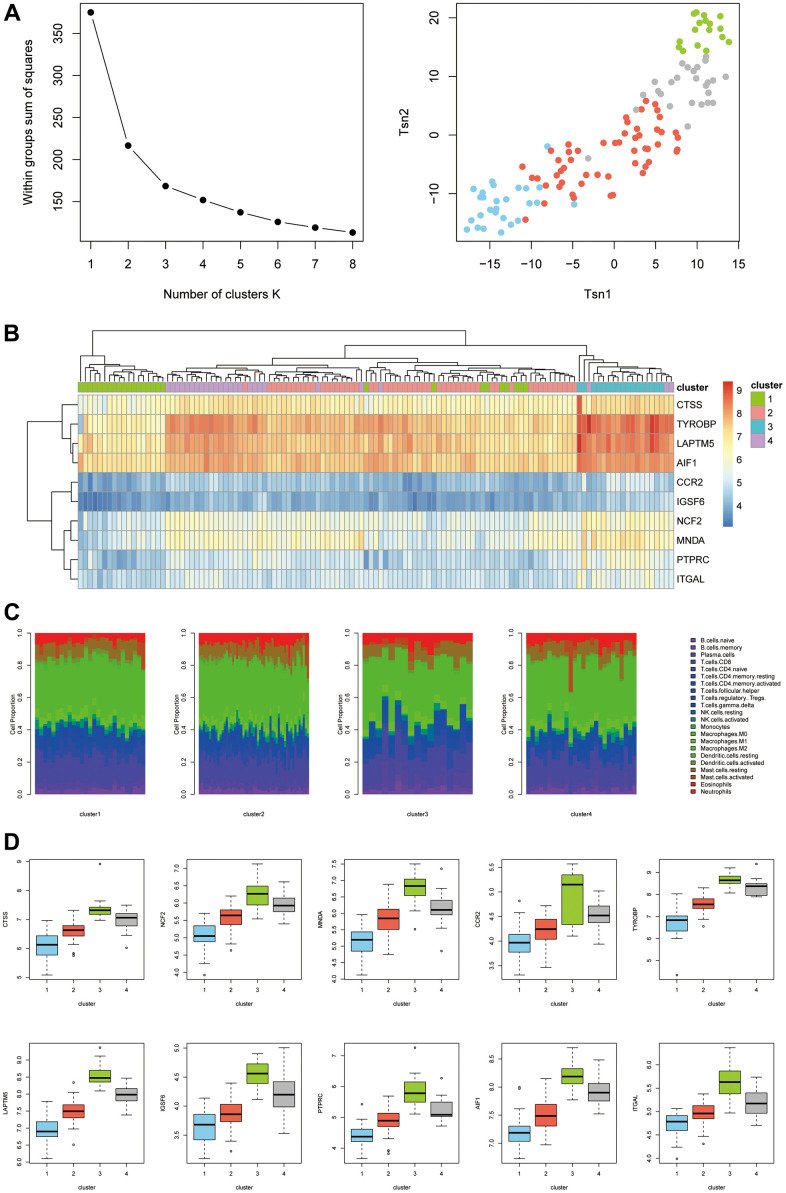
(**A**) K-means clustering classification. (**B**) Expression levels of different types of hub genes. (**C**) Differences in immune infiltration between different types. (**D**) Expression levels of hub genes in different types.

As shown in Figure 7A, we first determined the inflection point of SSE (i.e., the sum of the squares of the distances of all the points to the cluster center to which the points belonged). We searched for the optimal number of clusters K. When K=4, the SSE declined slowly, and all 123 samples were therefore classified into four categories by k-means clustering. Cluster 1 (blue), cluster 2 (red), cluster 3 (green), and cluster 4 (gray) had 27, 53, 17, and 26 samples, respectively (Figure 7A). The expression of the hub genes differed between clusters (Figure 7B). Therefore, the hub genes had important significance in disease classification. We next looked at the difference in immune infiltration of different types (Figure 7B). Cluster 1 and cluster 2 were significantly different from cluster 3 and cluster 4. We also examined the expression of hub genes in different types (Figure 7D) and found that the expression levels of all hub genes were ranked as cluster 1 < cluster 2 < cluster 4 < cluster 3.

## DISCUSSION

T lymphocytes play a central role in cell-mediated immunity. A large number of studies have shown that T cells are closely related to the occurrence of many types of cardiovascular diseases [22, 23], but their roles in AF pathogenesis have not been well established. Therefore, this study focused on T cells. Notably, however, the results of our analysis show that most DEGs are not strongly associated with T cells. Therefore, the hub genes identified in this study not only affect T cell function but may also have other functions. Because of the complexity and diversity of inflammatory responses during the occurrence and development of AF, we believe that AF samples should be classified using hub genes because these genes can more accurately guide exploration of the pathogenesis of AF and the development of relevant immunotherapy methods.

Of the 10 hub genes discovered in this study, *MNDA*, *TYROBP*, *LAPTM5*, *IGSF6*, *AIF1*, and *ITGAL* have never been reported to be associated with AF*.* We have now elaborated the possible mechanisms by which these genes are associated with AF.

*CTSS* encodes cathepsin S (CTSS), which is mainly involved in the pathogenesis of a variety of cardiovascular diseases by mediating the degradation of extracellular matrix (ECM) proteins [24]. Under ischemic and high-fat diet (HFD) loads, CTSS participates in cardiovascular remodeling and the formation of atherosclerosis by mediating transforming growth factor-beta (TGF-β) and peroxisome proliferator- activated receptor gamma (PPAR-γ) or activating the p38 mitogen-activated protein kinase (MAPK) pathway [25–27]. Cardiovascular remodeling, especially atrial reconstruction, is an important basis for the formation and maintenance of AF. Therefore, CTSS likely promotes the proliferation and differentiation of fibroblasts by influencing TGF-β and other related pathways, resulting in atrial reconstruction. However, this hypothesis still requires further experimental verification.

Neutrophil cytosolic factor 2 (NCF2), also known as NOXA2, is a subunit of NADPH oxidase. The function of this oxidase is to produce O2- using NADPH as a substrate. The change in the *NCF2* expression level significantly affects the level of reactive oxygen species (ROS), which is related to the occurrence and progression of cardiovascular diseases [28]. In addition, *NCF2* is mainly expressed in neutrophils, an important site for ROS production. Existing evidence suggests a significant causal relationship between neutrophil activity and the onset of AF [29]. A mouse-based study showed that fibrosis in AF relies heavily on neutrophil activation [30]. The production of ROS can activate metalloproteinases, a key enzyme family involved in fibrosis [31]. Whether NCF2 participates in the occurrence and progression of AF relying on the aforementioned mechanisms remains to be further explored.

*CCR2* encodes C-C motif chemokine receptor 2 (CCR2), which occurs ubiquitously on the surface of mononuclear macrophages. It binds CC motif chemokine ligand 2 (CCL2) and plays a major role in the recruitment of mononuclear macrophages to inflammatory sites [32]. The accumulation of macrophages is involved in TGF-β-mediated myocardial fibrosis. As a source of profibrotic-associated factors, macrophages can promote fibroblast proliferation and activation. Activated fibroblasts in turn promote structural remodeling of the atria by expressing fibrosis-related substances in large quantities [33, 34]. Macrophages also play an important role in electrical remodeling of the atria, which is mainly accomplished by their secretion of TNF-α. In summary, macrophages are involved in both the structural and electrical remodeling processes of the atria during the course of AF.

Myeloid nuclear differentiation antigen (MNDA) was first found to be expressed in macrophages and fibroblasts in inflammatory areas but not in noninflammatory areas. *MNDA* is highly expressed in macrophages in atherosclerotic plaques [35], but whether it is directly involved in the occurrence and progression of AF remains unknown. Given the role of macrophages in the occurrence and progression of AF, the inclusion of *MNDA* as a hub gene is presumably because MNDA is a characteristic marker of macrophages in inflammatory regions.

TYRO protein tyrosine kinase-binding protein (TYROBP) plays an important role in the pathogenesis of Alzheimer’s disease (AD). TYROBP seems to enhance the phagocytic activity of microglia, while timely clearing of apoptotic neurons and related metabolites is important for maintaining normal brain function. TYROBP can also mediate the anti-inflammatory response, thereby maintaining the immune balance function in the process of neuroinflammation. Triggering chronic inflammation results in neurodegenerative diseases, thereby increasing the risk of AD [36]. Interestingly, a series of clinical studies demonstrated that AF patients have a significantly increased risk of AD, but the cause of this phenomenon remains controversial [37–42]. Microglia in the brain rarely require peripheral supplementation in adulthood [43]. However, if microglial depletion occurs in the central nervous system under some special circumstances (e.g., inflammation, infection, injury, etc.), bone marrow–derived microglia are added [44]. AF patients may have chronic embolism or hemorrhage (macro- or micro), hypoperfusion, oxidative stress, and proinflammatory conditions [41]. Would this cause not only neuronal injury but also the depletion of microglia in the central nervous system? Is the change in the hub gene TYROBP also a hallmark of this pathological process? These questions remain to be further explored.

Allograft inflammatory factor 1 (AIF1) plays an important role in the occurrence and progression of atherosclerosis by stimulating the migration and proliferation of human smooth muscle cells and promoting the activation of macrophages [45]. AIF1 can induce the expression of fibrosis-related factors in normal fibroblasts [46]. AIF1 also induces monocytes to secrete IL-6 and enhance the chemotaxis of fibroblasts [47], thereby causing fibrosis. Given the important role of fibrosis in the occurrence and progression of AF, further exploring the potential mechanism of this gene during the occurrence and progression of AF would provide valuable data.

As a member of the protein tyrosine phosphatase (PTP) family, protein tyrosine phosphatase receptor type C (PTPRC) is also known as CD45 [48]. CD45 plays a role in regulating leukocyte adhesion, cytokine signaling, and immune receptor signaling (e.g., Fc, NK, Toll-like receptors) [48]. Accumulating evidence indicates that atrial tissue in AF patients is infiltrated with a large number of CD45^+^ cells [49–51]. Most of these CD45^+^ immune cells are CD68^+^ macrophages [52]. Given the important role of macrophages in the pathogenesis of AF, it makes sense that *PPTRC* would be a signature gene of AF. Integrin subunit alpha L (ITGAL), also known as CD11a, mainly functions through CD11a/CD18 integrins. It plays a role in the process of interleukocyte adhesion [53]. The role of CD11a in the pathogenesis of AF is unknown, but another integrin, CD11b/CD18, plays an important role in the pathogenesis of AF (ref?). The role of polymorphonuclear neutrophils (PMNs) in AF is mainly mediated by CD11b/CD18. The MPO released by PMNs directly activates more PMNs through the CD11b/CD18/MAPK pathway^58^, and the MPO released by PMNs furthermore acts directly on endothelial cells through cell junctions mediated by CD11b/CD18 integrins [54]. MPO can catalyze the oxidation of chloride to form hypochlorous acid, thereby activating MMP to participate in the atrial fibrosis process [29]. Whether CD11a/CD18 integrins play a similar role requires further investigation.

As a lysosome-related transmembrane receptor, lysosomal-associated protein transmembrane 5 (LAPTM5) is involved in the occurrence and progression of some neoplastic diseases [55]. Immunoglobulin superfamily member 6 (IGSF6) has been reported to be associated with inflammatory bowel disease [56]. Whether these two hub genes are involved in AF needs to be further explored.

## CONCLUSIONS

In this study, we identified the hub genes associated with AF/immune infiltration through a series of analytical methods and tools, including *CTSS*, *NCF2*, *MNDA*, *CCR2*, *TYROBP*, *LAPTM5*, *IGSF6*, *PTPRC*, *AIF1*, and *ITGAL*. The expression levels of these genes did not differ between samples from AF patients at different ages. However, the mRNA levels of *CTSS*, *IGSF6*, *CCR2*, and *PTPRC* were significantly higher in males than in females. We subsequently verified the above hub genes in an external dataset and then confirmed differences in AF/immune infiltration based on the screened hub genes. This study is the first to classify AF into four types using immune infiltration differences. Due to the small amount of sample data that could be included in this study, our results may not represent the full clinical picture of AF/immune infiltration. In addition, due to the difficulty of acquiring human atrial specimens, we cannot conduct biological validation on the results. Taken together, the findings of this study provide new insight into the pathogenesis and progression of AF from the perspective of immune infiltration.

## Supplementary Material

Supplementary Figures
