# Supplementary material for: Immune infiltration and immunophenotyping in atrial fibrillation
Source: Aging (Albany NY). 2023 Jan 4;15(1):213–29. doi: 10.18632/aging.204470 (PMC9876632; doi:10.18632/aging.204470)
Supplement: Supplementary Figures [file aging-15-204470-s001.pdf]

SUPPLEMENTARY FIGURES

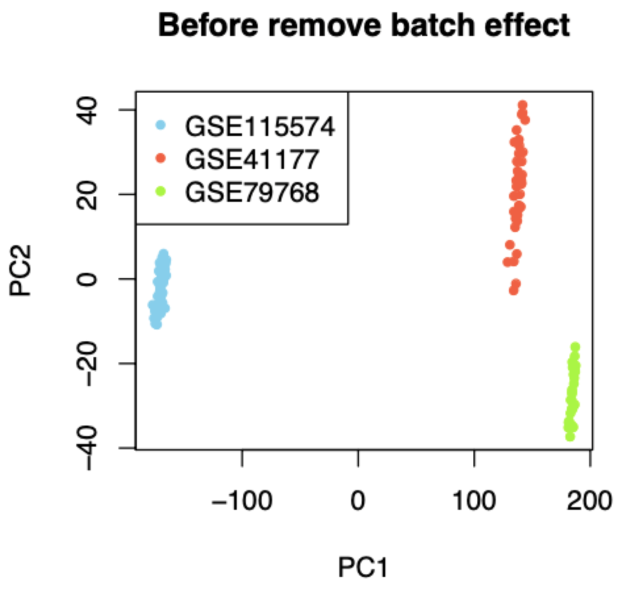

Supplementary Figure 1. The sample distribution.

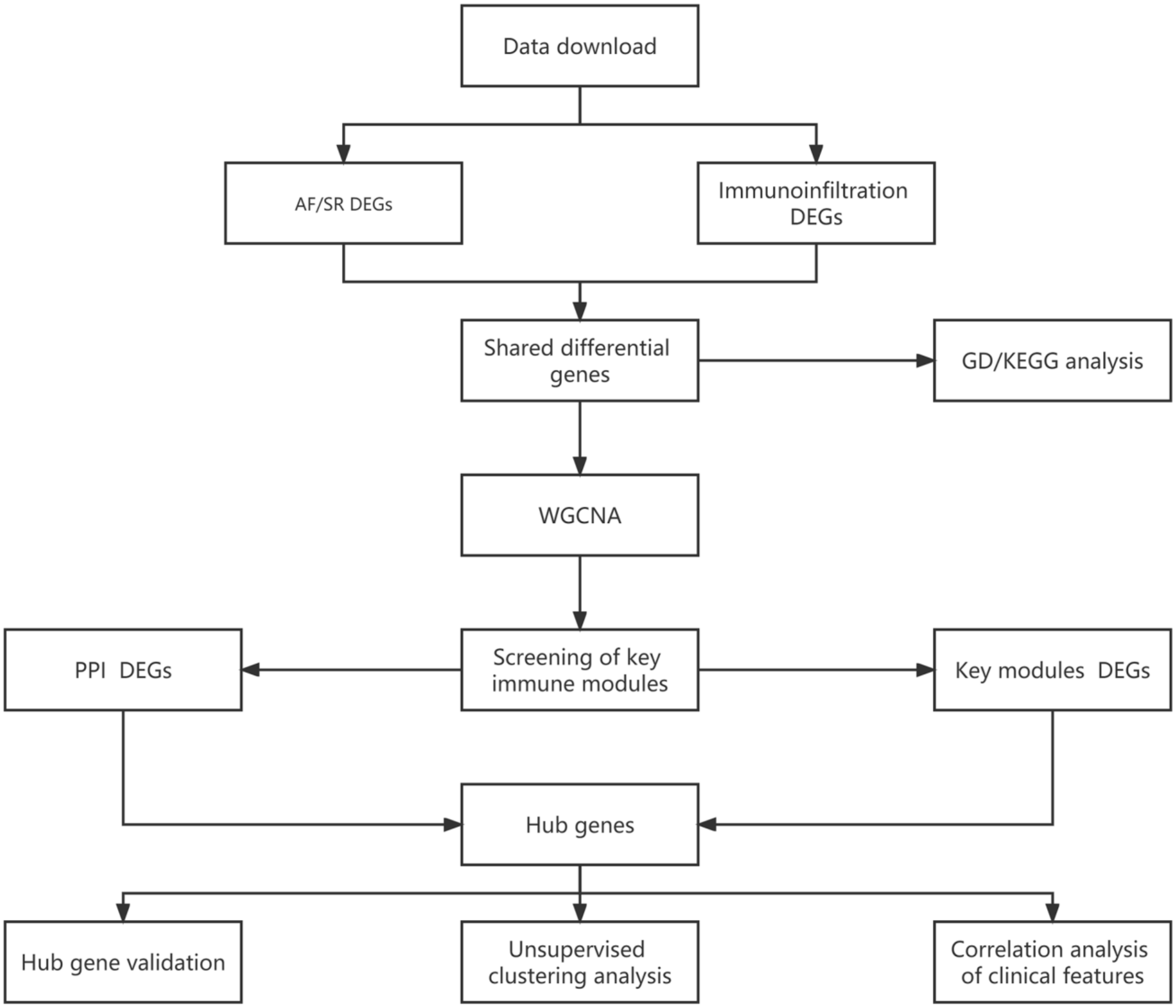

Supplementary Figure 2. Flowchart.
